# A novel polymer-free ciglitazone-coated vascular stent: *in vivo* and *ex vivo* analysis of stent endothelialization in a rabbit iliac artery model

**DOI:** 10.18632/oncotarget.11584

**Published:** 2016-08-24

**Authors:** Sylvia Otto, Kristin Jaeger, Frank D. Kolodgie, Diana Muehlstaedt, Marcus Franz, Sabine Bischoff, Harald Schubert, Hans R. Figulla, Renu Virmani, Tudor C. Poerner

**Affiliations:** ^1^ Department of Medicine 1, Division of Cardiology, University Hospital of Jena, Thuringia, Germany; ^2^ Institute for Laboratory Animal Science and Animal Protection (IVuT), University Hospital of Jena, Thuringia, Germany; ^3^ CV Path Institute Inc., Gaithersburg, MD, USA

**Keywords:** PPARg, ciglitazone, endothelium, vascular remodeling, drug-eluting stents, Pathology Section

## Abstract

**Aim:**

Peroxisome proliferator-activated receptor-gamma (PPARg) agonists have known pleiotropic cardiovascular effects with favourable properties in vascular remodeling, and specifically in suppression of vascular smooth muscle cell proliferation. A novel vascular stent coating using the PPARg ligand ciglitazone (CCS) was investigated regarding its effects on endothelialization after 7 and 28 days.

**Methods:**

Microporous bare metal stents (BMS) were coated with ciglitazone by ultrasonic flux with a load of 255 μg ciglitazone/stent. SixteenNew Zealand white rabbits, fed a with high cholesterol diet, underwent stent implantation in both iliac arteries. Everolimus-eluting stents (EES) and BMS were comparators. Histology (CD 31 immunostaining, confocal and scanning electron microscopy, morphometry) was performed after 7 and 28 days and by OCT (optical coherence tomography) in vivo after 28 days.

**Results:**

Microscopy showed comparable results with near complete endothelialization in CCS and BMS (%CD31 above stent struts after 7 days: 67.92±36.35 vs. 84.48±23.86; *p* = 0.55; endothel % above stent struts: 77.22±27.9 vs. 83.89±27.91; *p* = 0.78). EES were less endothelialized with minimal fibrin deposition, not found in BMS and CCS (% CD 31 above struts after 28 days, BMS: 100.0±0.0 vs. EES: 95.9±3.57 vs. CCS: 100.0±0.0; *p* = 0.0292). OCT revealed no uncovered struts in all stents after 28 days.

**Conclusions:**

Polymer-free coating with ciglitazone, a PPARg agonist is feasible and stable over time. Our data prove unimpaired endothelial coverage of a ciglitazone-coated vascular stent system by histology and OCT. Thus, this PPARg agonist coating deserves further investigation to evaluate its potency on local neointimal suppression.

## INTRODUCTION

Thiazolidinedione, also known as peroxisome proliferator-activated receptor-gamma (PPARg) agonists, have been clinically introduced as oral antidiabetics in type 2 diabetes mellitus [1]. Peroxisome proliferator-activated receptors show multiple pleiotropic effects due to their modulation of many target genes involved in cell growth, proliferation, differentiation and inflammation. PPARg are expressed in smooth muscle cells and endothelial cells, and are predominantly found in adipose tissue. Previous animal studies and smaller clinical investigations showed promising anti-atherosclerotic and antiproliferative effects of systemically administered PPARg agonists [2–5]. More specifically, PPARg activation can attenuate atherosclerosis and mediates cholesterol efflux [6, 7]. Multiple preclicinical studies have proven the favourable effects of PPARg stimulation in the process of vascular and cardiac remodeling by prevention of smooth muscle cell proliferation and migration via various pathways [7–12]. Ciglitazone is a ligand of PPARg, which also acts as an antiangiogenic agent [13]. Vascular drug-eluting stents (DES) with a metallic or bioresorbable backbone have proven superiority compared to bare metal stents (BMS) with respect to long-term patency and reduced restenosis rates [14]. This benefit is partially offset by delayed endothelial healing requiring prolonged dual antiplatelet therapy [15, 16]. Despite modern advancements in device technology, such as new generation DES, bioresorbable vascular scaffolds (BVS) or drug-coated balloons, the important issues of persistent peri-stent inflammation and development of neoatherosclerosis are remaining [17]. Thus, the ideal antiproliferative substance, allowing fast endothelialisation simultaneously to maximal inhibition of neointimal proliferation, is still undiscovered. Therefore, we further investigated PPARg agonists, as an alternative substance class possibly allowing a favourable course of vascular remodeling.

### Aims of the Study

We aimed to prove the concept of a novel ciglitazone-coated stent (CCS) in terms of (1) feasibility of polymer-free ciglitazone stent coating, and (2) unimpaired stent endothelialisation after 7 and 28 days, investigated ex vivo by histology and *in vivo* by optical coherence tomography (OCT) in an animal model.

## RESULTS

### UV-Vis spectroscopic analysis of stent coating

Photometric determined ciglitazone content after coating of stent systems was 255.15 μg ± 11.95 ciglitazone per stent system corresponding to ~ 21.3 μg/mm stent length. The same coating content was measured after storing stent systems for 4 months at ~ 4 - 7°C. UV-Vis spectra of extracted stent systems and standard solutions were identically after one extraction. HPLC analyses of stent systems after stent deployment *in vivo* obtained a mean ciglitazone content of 8.80 ± 1.48 μg per balloon, which is equivalent to 3.45 ± 0.58 % of the initial coating content.

### Microscopic analysis after 7 days

Scanning electron microscopy and confocal microscopy after CD31 staining showed comparable results between CCS stents and BMS, and a fairly well progressed endothelialization seven days after stent implantation (Table 1).

**Table 1:** Quantification of 7-Day endothelialization by confocal and electron microscopy

Microscopy	CCS (*n*= 3)	BMS (*n*= 3)	*p* value
**Confocal results (CD31 staining)**			
%CD31 above stent struts	67.92±36.35	84.48±23.86	0.55
%CD31 between stent struts	68.79±24.55	85.00±24.55	0.58
**Scanning electron microscopy results**			
Endothel % above stent struts	77.22±27.91	83.89±27.91	0.78
Endothel % between stent struts	87.22±20.21	88.33±20.21	0.95

BMS, bare metal stent; CCS, ciglitazone-coated stent

### Endothelial coverage by En Face SEM after 28 days

All devices appeared widely and evenly expanded in x-ray evaluation without evidence of stent fractures.

**Figure 1:**
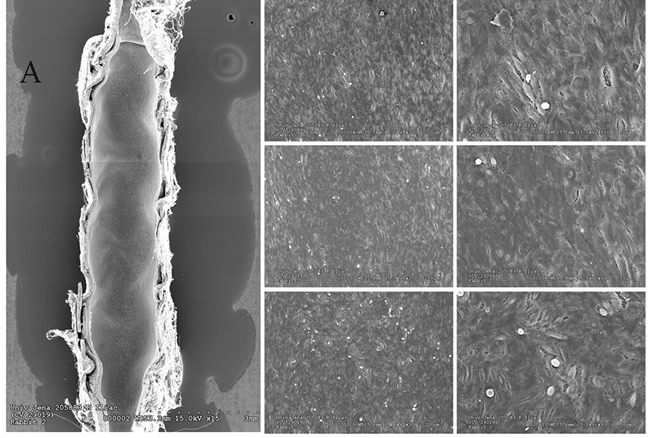

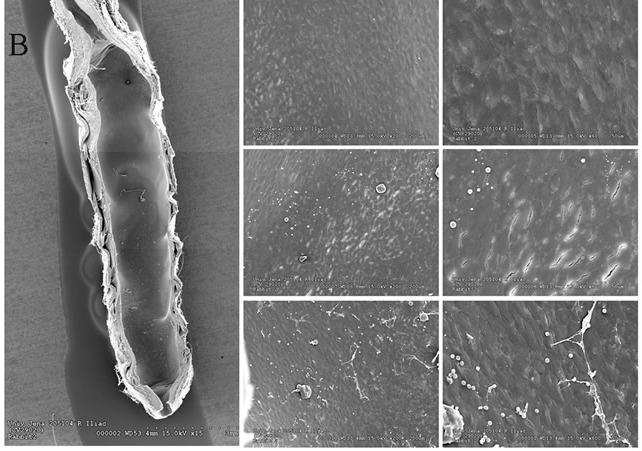

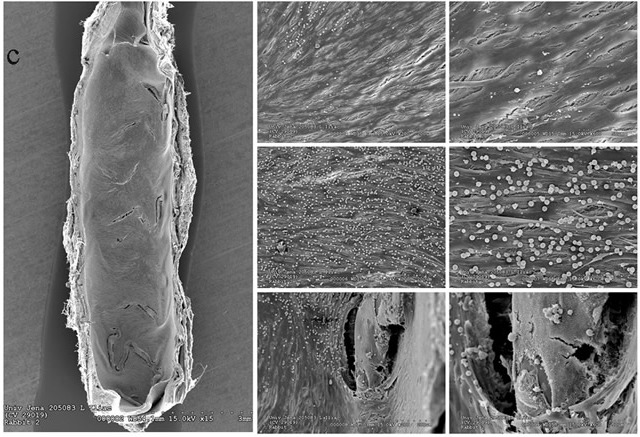
Representative low (x15) and higher power (x200, x600) scanning electron microscopy images of 28-day implanted ciglitazone-coated stents in the iliofemoral arteries of hypercholesterolemic rabbits Higher power magnifications are from proximal (top panels), mid (middle panels), and distal regions (bottom panels) of the stent. Left: En face view of the stent surface shows barely visible struts underlying a thin layer of neointima completely covered by endothelium. Note the luminal surface is smooth. Middle: Higher power views shows spindle to cobblestone-shaped endothelial morphology with a few adherent inflammatory cells, particularly observed in the distal region. Right: Monolayer of spindle-shaped endothelial cells in close apposition with occasional adherent inflammatory cells. **Figure 1B:** Representative low (x15) and higher power (x200, x600) scanning electron microscopy images of 28-day implanted bare metal stents (BMS) in the iliofemoral arteries of hypercholesterolemic rabbits. Higher power magnifications are from proximal (top panels), mid (middle panels), and distal regions (bottom panels) of the stent. Left: En face view of the luminal surface shows a relatively thickened layer of neointima where underlying stent struts are essentially obscure. Note the luminal surface is smooth. Middle: Higher power views show spindle to cobblestone-shaped endothelial morphology with rare residual red blood cells and adherent platelets, particularly observed in the distal region. Right: Monolayer of spindle to cobblestone-shaped endothelial cells aligned in the direction of flow and in close apposition. **Figure 1C:** Representative low (x15) and higher power (x200, x600) scanning electron microscopy images of 28-day implanted everolimus-eluting stents (EES) in the iliofemoral arteries of hypercholesterolemic rabbits. Higher power magnifications are from proximal (top panels), mid (middle panels), and distal (bottom panels) regions of the stent. Left: En face view of the luminal surface shows a relatively thin layer of neointima overlying stent struts, which are partially exposed in the distal segment (bottom). Middle: Higher power views shows a spindle-shaped endothelial morphology with collections of adherent monocyte/macrophages, particularly seen in the middle panels. Right: Monolayer of loosely apposed spindle-shaped endothelial cells with adherent monocytes/macrophages. The lower right panel represents a high power view of an exposed stent strut covered by a thin layer of platelet thrombus with a few adherent inflammatory cells.

Representative SEM images of implanted stent types for 28 days are displayed in Figure 1A-1C. The estimated endothelial coverage of luminal surface was 100 % for most CCS and BMS and > 70 % for most EES. A few EES (3 of 6) showed evidence of delayed healing exhibiting occasional uncovered struts with adherent platelets and inflammatory cells.

### CD31 immunostaining after 28 days

Stents from all three groups showed strong expression of CD31 over the majority of stent surfaces indicating mature endothelial cells with good cell-to-cell apposition. However, there were several regions of artefacts, which inaccurately reflected lower and significantly different % endothelial scores between groups (Table 2). After exclusion of vessels with artefacts, EES revealed significantly lower percent endothelialisation above stent struts (*p* = 0.0292) and a trend toward lower endothelialisation between struts (*p* = 0.0585) as compared to CCS and BMS (Table 2).

**Table 2:** Visual estimation of 28-day stent endothelialization by confocal imaging of CD31-staining

CD31	CCS *n*= 6	EES *n*= 6	BMS *n*= 6	*p* value
**Above struts, %**	89.26±26.31	61.57±45.91	72.31±34.88	0.438
**Between struts, %**	89.26±25.23	62.69±45.89	70.65±37.09	0.462
	**After excluding artifacts**			
	***n* = 5**	***n* = 3**	***n* = 3**	
**Above struts, %**	100.0±0.00	95.93±3.57	100.0±0.00	0.0292
**Between struts, %**	99.56±0.72	96.30±3.39	100.0±0.00	0.0585

BMS, bare metal stent; CCS, ciglitazone-coated stent; EES, everolimus-eluting stent.

### Morphometry after 28 days

Tables 3 and 4 display the results from the histomorphometric analysis. None of the struts in either groups were malapposed. Minimal fibrin deposition was only seen in EES with rare fragments present in two BMS sections and in none of the CCS stents (Table 3). Endothelialization was essentially complete in both CCS (99.83 ± 0.28 %) and BMS (98.30 ± 4.16 %) with significantly lower values in EES (87.35 ± 11.87; *p =* 0.0185; Table 2). Less inflammation, primarily observed as macrophage infiltration, was found in EES compared to CCS and BMS (*p* = 0.0002; Table 4).

**Table 3:** Histologic ANOVA comparison of vessel injury and healing, 28-days after stent implantation

Stent Group			Struts with Fibrin (%)	Fibrin Score	Malapposition (%)	Struts with RBCs (%)	Endothelial coverage (%)	Mineralization (%)
Cigli	*n* = 6		0.00±0.00	0.00±0.00	0.0±0.0	2.86±5.71	99.83±0.28	1.59±2.46
EES	*n* = 6		12.25±17.31	0.17±0.28	0.0±0.0	2.96±5.05	87.35±11.87	4.74±6.93
BMS	*n* = 6		1.59±2.46	0.00±0.00	0.0±0.0	5.31±9.80	98.30±4.16	3.17±3.89
***P* value**		**All groups**	0.106	0.120	-	0.802	**0.0185 CCS & BMS vs. EES**	0.539
		**CB (I)**	0.374	1.00	-	0.327	0.158	0.678
		**EC (II)**	0.374	0.317	-	0.918	0.108	0.462
		**BE (III)**	0.350	0.317	-	0.688	0.233	0.675

CB, ciglitazone-coated stents (CCS) vs. bare metal stents (BMS); EC, everolimus-eluting stents (EES) vs. ciglitazone-coated stents; BE, bare metal stents vs. everolimus-eluting stents; RBCs, red blood cells.

Analysis includes mean of all sections.

**Table 4:** Histologic ANOVA comparison of inflammatory response 28-days after stent implantation

Stent Group			Struts with Granulomas (%)	Inflammation Score	Adventitial Inflammation Score	Struts with Giant Cells (%)
Cigli	*n* = 6		0.60±1.46	1.41±0.47	0.19±0.31	13.28±15.89
EES	*n* = 6		0.00±0.00	0.14±0.17	0.11±0.17	12.58±11.03
BMS	*n* = 6		0.00±0.00	1.61±0.66	0.39±0.65	8.69±7.77
***P* value**		**All groups**	0.391	**0.0002CCS & BMS vs. EES**	0.759	0.779
		**CB (I)**	-	0.637	0.796	0.917
		**EC (II)**	0.374	**0.0463**	0.814	0.757
		**BE (III)**	-	**0.0495**	0.121	0.916

CB, ciglitazone-coated stents vs. bare metal stents; EC, everolimus-eluting stents vs. ciglitazone-coated stents; BE, bare metal stents vs. everolimus-eluting stents; RBCs, red blood cells.

Analysis includes mean of all sections.

### *In vivo* evaluation by OCT after 28 days

**Figure 2:**
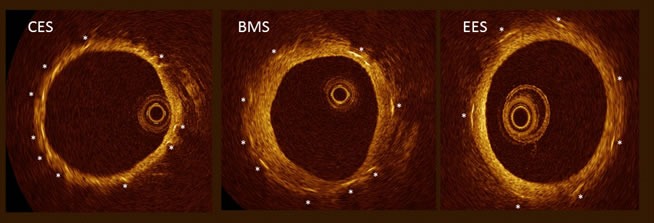
Representative OCT images of ciglitazone-coated stents (CES), bare metal stents (BMS)and everolimus-eluting stents (EES) 28 days after in implantation, asterixis indicate stent struts

None of the stent struts were malapposed accounting for well-sized and implanted stents at the index procedure. There were no differences between CCS and BMS regarding stent coverage (Figure 2). Almost all stents were fully contained within the vessel wall (embedded: 100 % CCS struts vs. 98.3 ± 0.17 EES struts vs. 99.3 ± 1.04 BMS struts, n.s.; apposed and covered: 0 % CCS struts vs. 1.24 ± 0.47 EES struts vs. 0.74 ± 1.04 BMS struts, n.s.). No uncovered struts were observed.

## DISCUSSION

To the best of our knowledge, we investigated for the first time ciglitazone as an alternative to common antiproliferative coating substances for vascular stent systems.

Polymer free coating with a predefined ciglitazone content is feasible. Stent coating was stable over a tested storage period of four months. Furthermore, more than 96 % of the ciglitazone coating was released *in vivo* after single balloon inflation. We specifically investigated ciglitazone out of the substance class of thiazolidinediones since it might exert stronger and additional PPARg-independent antiproliferative effects compared to the oral antidiabetic agent rosiglitazone. [18, 19] Moreover, pioglitazone is not suitable for stent coating due to its lipophilic structure.

We investigated endothelial coverage of the implanted stents ex vivo with different methods: (1) en face SEM, (2) immunostaining with CD31, and (3) histomorphometry; and at two different time points (7 and 28 days). Despite some artifacts, endothelial coverage was unimpaired and nearly 100 % in CCS revealing comparability with BMS. More importantly, our data indicate better endothelialization of CCS compared to EES in this rabbit model 28 days after stent implantation. The microscopic data were underlined by *in vivo* OCT evaluation, which also showed nearly completed in wall stent incorporation after 28 days with no differences between CCS and BMS. In addition, microscopic well-advanced endothelialization of CCS as early as seven days after stenting comparable to BMS has been demonstrated. Previous animal and human autopsy studies could show a corresponding, but deferred arterial healing response after stent implantation in human coronary arteries compared to animal iliacal arteries [20]. Thus, our data suggest safety of an alternative ciglitazone coated stent *in vivo* in terms of unaltered endothelialization. EES revealed less inflammation due to macrophage autophagy as previously reported [21, 22].

Morphometric analysis in this study revealed no fibrin deposition on CCS stents compared to EES and even to BMS. Fibrin is found early after stent placement and its associations with neointimal growth and neoatherosclerosis has been shown repeatedly [23]. Fibrin deposition products seem to also act as a direct stimulus for smooth muscle cell (SMC) migration and are accompanied by platelets, which serves as an attributable risk for ST [24]. This study was intended to prove the concept of polymer-free ciglitazone coating of a vascular stent system and its safety *in vivo*. The absence of fibrin and a near complete endothelial stent coverage account for a favorable interaction between ciglitazone, the metallic cage and arterial vessel wall. Our data are in line with a previous study suggesting accelerated endothelialization after arterial denudation in rats orally treated with troglitazone, another PPARg agonist [25]. Furthermore, there is growing evidence for the antiproliferative and anti-inflammatory properties of PPARg agonists from ex vivo, animal and human studies. PPARg agonists decrease expression of matrix metalloproteases (MMPs), suppress macrophage activity and inhibit migration and proliferation of vascular SMCs [8–12, 26–28]. Also, inhibitory effects on neointimal hyperplasia, reduction of restenosis rates and preservation of vein graft intergrity have been demonstrated in various preclinical and clinical studies after systemical administration of PPARg [2, 29–31]. Given these beneficial effects and the safety results of this study, we believe it is worth further investigating local application of PPARg agonists as a novel stent coating or even for pre-treatment of endovascular grafts.

### Clinical implications

Ciglitazone might be an interesting alternative to conventional limus drugs for drug-eluting vascular devices allowing both, fast stent endothelialisation and neointimal suppression. Pharmacokinetic studies need to identify the optimal drug load of ciglitazone. Additional efficacy studies in swines, which is a better model for proliferation must further investigate the antiproliferative strength of a local ciglitazone application before clinical tests in humans can be planned.

### Limitations

The high proportion of artifacts limiting confocal microscopy after immunostaining has to be disclosed. OCT cannot precisely differentiate the quality of stent coverage (endothelium versus fibrin deposits). However, OCT imaging in this study was performed additionally to extended histological analysis, which was used for validation of endothelial coverage. Moreover, OCT images of the different investigated stents give a first impression for their antiproliferative strength in this animal model.

## CONCLUSIONS

Polymer-free stent coating with ciglitazone, a PPARg agonist is feasible and stable over time. Our data prove unimpaired endothelial coverage of a ciglitazone-coated vascular stent system by histological analysis ex vivo and by OCT *in vivo*. Thus, this novel stent coating deserves further investigation to evaluate its potency on neointimal suppression.

## MATERIALS AND METHODS

### Study design

The novel ciglitazone-coated stent (CCS) was tested *in vivo* in two small test series in a total of 16 rabbits (Table 5). Rabbits were fed a high cholesterol diet, and then underwent stent implantation in both common iliac arteries via carotid artery. Commercially available bare metal stents (BMS, Yukon Choice 4^®^, Translumina, Hechingen, Germany) and everolimus-eluting stents (EES, Xience V^®^, Abbott Vascular, Santa Clara, USA) were used as comparators. Stented vessel segments were extracted after 7 or 28 days and processed for post-mortem histological analysis. In three animals stent coverage was assessed *in vivo* using OCT (M2 CV system, LightLab Imaging Inc., Westford, MA, USA) for intravascular, high-resolution imaging as previously described [32].

**Table 5:** Implantation matrix and study design

Animal Number	STENT Type		Combination	Evaluation	Time Frame
	Left Iliac	Right Iliac			
Series I					
1	EES	CCS	EC (II)	Histology	28 days
2	EES	CCS	EC (II)	Histology	28 days
3	CCS	BMS	CB (I)	Histology	28 days
4	CCS	BMS	CB (I)	Histology	28 days
5	BMS	EES	BE (III)	Histology	28 days
6	EES	CCS	EC (II)	Histology	28 days
7	CCS	BMS	CB (I)	Histology	28 days
8	BMS	EES	BE (III)	Histology	28 days
9	BMS	EES	BE (III)	Histology	28 days
10	CCS	BMS		OCT	28 days
11	BMS	EES		OCT	28 days
12	EES	CCS		OCT	28 days
Series II					
13	CCS	BMS		Histology	7 days
14	BMS	CCS		Histology	7 days
15	CCS	BMS		Histology	7 days
16	BMS	CCS		None*	7 days

BMS, bare metal stent; CCS, ciglitazone-coated stent; EES, Xience V everolimus-eluting stent;

* animal died due to fatal infection before study termination.

### Polymer-free coating

A spray solution of 3.4 mg/ml was prepared after dissolving ciglitazone in ethanol. Microporous BMS (Yukon Choice 4^®^, Translumina, 2.5 or 3.0 × 12 mm) were coated with ciglitazone by ultrasonic flux (MediCoat Stent Coating System, SonoTek^®^) with a drug load of 255 μg ciglitazone/stent (225 μg/cm stent length).

### UV-Vis spectroscopy of coating substance

The ultraviolet-visible (UV-Vis) spectra of the ciglitazone test solution and standard solution were obtained with a spectrophotometer. Coated stent systems were extracted in 6 ml ethanol by an ultrasonic bath of 15 minutes after coating, and also after storing over a period of 4 months. Completeness of coating dissolution was verified with re-extraction of the remaining stent system. All wavelength scans were performed at room temperature. The UV-Vis absorption of the test solution was measured at 228 nm. Absorbance spectra were also measured at 350 and 200 nm to prove purity of the test solution. The absorbance of ethanol solution (1μg/ml) was 0.0467 and the content of coating was calculated. After stent deployment in rabbits the remaining stent system was extracted in ethanol and filtered. High-performance liquid chromatography (HPLC) was conducted with a RP18 separation system (UV-Vis detection at 228 nm) and possibly remaining ciglitazone was measured.

### Animals

This study was conducted at the Institute for Laboratory Animal Science and Animal Protection (IVuT) at the University Hospital of Jena according to the German Protection of Animal Act. The study protocol was approved by the appropriate State Office of Food Safety and Consumer Protection (TLLV, Bad Langensalza, Thuringia; local registration number: 02-028/07). New Zealand White rabbits were fed an atherogenic Western diet consisting of 1 % cholesterol 14 days prior stent implantation and consisting of 0.15% cholesterol for the remaining study time.

### Surgical procedure

Rabbits were anesthetized and access of the carotid artery was surgically prepared. Endothelial denudation of both common iliac arteries was conducted using an angioplasty balloon catheter. Subsequently, animals received left and right iliac stents. Stents (2.5 × 12 mm in series I, and 3,0 × 12 in series II) were implanted achieving moderate oversizing (~ 1.3:1 ratio). A single dose of heparin (150 IU/kg) was given during catheterization. Carotid artery was ligated at the end of the procedure. Rabbits were anticoagulated by daily administration of aspirin (12 mg / kg) and clopidogrel (4 mg / kg). At follow-up, animals were euthanized and the distal aorta to the proximal femoral arteries were dissected after exsanguination by flushing heparinized Ringer's lactate followed by 10 % neutral buffered formalin.

### Optical coherence tomography

In study series II, three animals (#10-12) were examined *in vivo* by OCT: After surgical preparation of the contralateral carotid artery, the OCT image wire was advanced to the iliac stents, and OCT imaging was conducted by proximal balloon inflation and saline flushing to establish local exsanguination during imaging. OCT analysis was conducted offline by two investigators (K.J. & D.M.) blinded to the stent type. Analysis of stent cross sections was performed at maximum of 1 mm intervals or frame by frame, if irregularities were observed. Stent struts were counted and classified as embedded (contained within the vessel wall); apposed and covered, if tissue was seen above the strut; apposed and uncovered, if no tissue was detected; or malapposed [33].

### Histological evaluation

#### Scanning electron microscopy and morphometric analysis

Stented iliac arteries were bisected longitudinally to expose luminal surface. Half of the stents were processed for scanning electron microscopy (SEM). En face SEM images were taken at low power (x 15 magnification) to visually estimate neointimal incorporation of the entire luminal stent surface. Images were incrementally enlarged (x 50, x 200 and x 600) and percent endothelial coverage was visually estimated.

Stented segments were also evaluated by light microscopy and analyzed with the morphometry software (IP Lab for Mac OS X, Scanalytics, Rockville, MD). Each stent section was analysed for strut apposition, fibrin deposition, granuloma reactions, mineralization and haemorrhage. Percentages were calculated in relation to the total number of struts in each section. Endothelial surface coverage above and between stent struts were semi-quantified and expressed as the percentage of the lumen circumference covered by endothelium. The percent endothelialization was downgraded if loose intercellular junctions with adherent platelets and/or inflammatory cells were observed.

#### Immunostaining and confocal imaging

The opposite sides of the bisected stent halves were incubated with antibody markers for CD31 (1:20 dilution, Dakom Carpenteria, California). Specific binding was visualized by the secondary antibody donkey anti-mouse Alexa Flour 488 (1:150 dilution, Invitrogen Corp., Carlsbad, California). TOTO-3 iodide was used as a nuclear counter-stain (1:100 dilution). The specimens were mounted en face on glass slides and representative images were acquired (Zeiss™ Pascal laser confocal microscope). The percentage of CD31 staining was visually estimated along the entire stent (above and between stent struts) by conventional fluorescence microscopy. The endothelial cell marker CD31, also known as platelet endothelial cell adhesion molecule (PECAM-1), was identified as localized expression at the cell's periphery indicating mature intercellular junctions. Non-stented proximal arterial segments served as positive controls.

### Statistical analysis

All calculations were done using SPSS™ for Windows (Version 19.0, IBM™ SPSS Statistics) and JMP (Version 5.0, Cary, NC). Data are expressed as means ± standard deviation (SD). Continuous variables were compared between groups using one-way analysis of variance (ANOVA). Non-parametric histological scores were compared using Wilcoxon/Kruskal-Wallis rank sums tests. Statistical significance was considered for a *p* - value < 0.05. Additional post-hoc analysis was performed by Tukey-Kramer, if statistical significance was shown.
